# An Application of Bayesian Approach in Modeling Risk of Death in an Intensive Care Unit

**DOI:** 10.1371/journal.pone.0151949

**Published:** 2016-03-23

**Authors:** Rowena Syn Yin Wong, Noor Azina Ismail

**Affiliations:** Department of Applied Statistics, Faculty of Economics and Administration, University of Malaya, Kuala Lumpur, Malaysia; Azienda Ospedaliero-Universitaria Careggi, ITALY

## Abstract

**Background and Objectives:**

There are not many studies that attempt to model intensive care unit (ICU) risk of death in developing countries, especially in South East Asia. The aim of this study was to propose and describe application of a Bayesian approach in modeling in-ICU deaths in a Malaysian ICU.

**Methods:**

This was a prospective study in a mixed medical-surgery ICU in a multidisciplinary tertiary referral hospital in Malaysia. Data collection included variables that were defined in Acute Physiology and Chronic Health Evaluation IV (APACHE IV) model. Bayesian Markov Chain Monte Carlo (MCMC) simulation approach was applied in the development of four multivariate logistic regression predictive models for the ICU, where the main outcome measure was in-ICU mortality risk. The performance of the models were assessed through overall model fit, discrimination and calibration measures. Results from the Bayesian models were also compared against results obtained using frequentist maximum likelihood method.

**Results:**

The study involved 1,286 consecutive ICU admissions between January 1, 2009 and June 30, 2010, of which 1,111 met the inclusion criteria. Patients who were admitted to the ICU were generally younger, predominantly male, with low co-morbidity load and mostly under mechanical ventilation. The overall in-ICU mortality rate was 18.5% and the overall mean Acute Physiology Score (APS) was 68.5. All four models exhibited good discrimination, with area under receiver operating characteristic curve (AUC) values approximately 0.8. Calibration was acceptable (Hosmer-Lemeshow p-values > 0.05) for all models, except for model M3. Model M1 was identified as the model with the best overall performance in this study.

**Conclusion:**

Four prediction models were proposed, where the best model was chosen based on its overall performance in this study. This study has also demonstrated the promising potential of the Bayesian MCMC approach as an alternative in the analysis and modeling of in-ICU mortality outcomes.

## Introduction

In most developed countries, prognostic models are commonly used to predict mortality outcomes for critically ill patients in the intensive care unit (ICU). Development of these predictive models usually involved the use of logistic regression approach. As these models are based on formal probabilistic reasoning, they offer an objective approach in predicting mortality outcomes and provide results that are reproducible over time. These models are useful tools in aiding clinicians in decision making, interpretation of diagnosis and prescription of appropriate treatment options to patients [1]. They also assist hospital administration in making planned changes in resource allocation, such as adjustments in staffing ratios and ICU bed number [2]. More importantly, an accurate and reliable prognostic model can be used for benchmarking purposes to compare the quality and clinical performances of different ICUs.

Advances in computing power have supported the development of newer generations of ICU prognostic models such as the Acute Physiology and Chronic Health Evaluation (APACHE) IV [3], SAPS 3 Admission Score Model [4–5] and MPM_0_-III admission model [6]. Although they are popular in developed nations such as the United States, Europe and Australia, application of these models are not that widespread in developing countries in South East Asia. The use of automation and information technology is required to support the extensive data collection process and increased complexity of the latest models. Most ICUs in the developing countries do not have the technological advance that is conducive for application of the latest prognostic models due to constraints in costs, infrastructures and resources.

In Malaysia, most ICUs are still following the practice of manual data collection as they are not equipped with automated patient monitoring systems. These ICUs participate on a voluntary basis in annual national audits that are conducted by the Malaysian Registry of Intensive Care (MRIC). Evaluation of the annual performances of participating ICUs is performed through a comparison of SAPS II [7] severity of illness scores. The ICUs are ranked according to their performances in terms of SAPS II scores and outcomes of the audits are officially declared in annual reports [8]. SAPS II scores are used as the benchmark in the national audits because the parameters in SAPS II are easily available in all ICUs, including those at the district level. A limitation of this assessment is that the predictive component of SAPS II model is not used in the reporting of ICU performance in the national audits, and assessment of ICU performance is entirely based on SAPS II scores.

There is a lack of research in ICU prognostic modeling in Malaysia. In our earlier study [9], external validation of APACHE IV in a Malaysian ICU revealed that APACHE IV had good discrimination power but poor calibration. APACHE IV overestimated in-ICU mortality risk, especially for mid to high risk patient groups. The model's lack of fit was due to differences in patient management and case mix between APACHE IV and the Malaysian ICU.

The primary objective of this study is to develop and propose suitable prognostic models for application in a Malaysian ICU. We also attempt to investigate the significant factors that affect mortality risk in a Malaysian ICU. However, instead of using the conventional maximum likelihood approach, we apply the Bayesian Markov Chain Monte Carlo (MCMC) approach as an alternative in the modeling of ICU risk of death in a Malaysian ICU.

There are numerous advantages in employing a Bayesian approach in developing ICU prognostic models. The Bayesian approach considers data to be fixed and assumes parameters as random variables, where the uncertainty of unknown parameters is taken into consideration via probability theory. This provides a degree of uncertainty in the model, yielding predictions that are more realistic and safeguards against overfitting of models more than frequentist approaches. Moreover, the Bayesian approach relies on exact inference, instead of large sample asymptotic approximations. This facilitates an easier and more intuitive interpretation of the credible intervals of the estimated parameters of a predictive model. The flexibility of the Bayesian approach to update prior information about the underlying parameters with information from cumulative or past experience is also considered one of its advantages [10].

Despite these advantages, the Bayesian approach is not so popular because it is often computationally intensive, especially for models that involve many variables. Its application requires the use of specialized software and the knowledge to perform MCMC analyses. As such, the Bayesian approach is underutilized in areas of prognostic modeling for general ICU mortality outcomes. Although there were several studies that applied Bayesian MCMC for prediction of in-hospital risk of death, their areas were limited to specific subgroups of patients with diseases such as trauma [11], cancer and AIDS [12], acute myocardial infarction [13] and malaria [14]. These studies were mostly focused on application of Bayesian MCMC approach in variable selection and model choice. In this study, the Bayesian MCMC approach was used for identification of significant risk predictors and estimation of model parameters.

## Materials and Methods

### Data collection

This prospective study involved a cohort of 1,286 critically ill patients who were admitted to the Hospital Sultanah Aminah Johor Bahru (HSA) ICU between January 1, 2009 and June 30, 2010. The single multidisciplinary ICU in HSA is equipped with sixteen beds and provides services to general medical, surgical and trauma patients. Post-coronary artery bypass graft (CABG) patients were excluded from the study because these patients receive treatment in a separate unit in the hospital. Patients who were below 16 years of age, those who were transferred from another ICU/hospital, with less than 4 hours of ICU stay, as well as, patients who were seeking treatment for burns and transplant procedures were excluded from analysis. Data from the first admission was used for patients with multiple admissions. A total of 1,111 patients met the inclusion criteria and were considered for the study.

This study was approved by the Medical Research and Ethics Committee, Ministry of Health, Malaysia. The requirement for informed consent from all participants was waived because data collection was based on existing medical and laboratory records and there was no clinical intervention in this study. Patient records or information were anonymized and de-identified prior to analysis. Routine data collection was manually performed by HSA ICU nurses, and then manually transferred to an online database by the medical officers. Individual user accounts were created for each of the data entry personnel in order to preserve data integrity and traceability.

Data collected at the time of ICU admission included socio-demographic (age, gender and ethnicity), admission (date and time of admission, source and operative status) and discharge details (vital status and discharge location). The APACHE IV approach was adopted in collection of data in this study. Although variables that were defined in APACHE IV were collected, the frequency of data collection followed the current practice of HSA ICU. Information on patients' existing chronic health conditions were categorized into eight co-morbidities (AIDS, metastatic cancer, cirrhosis, hepatic failure, immunosuppression, leukemia or myeloma, lymphoma and diabetes). The main reason for ICU admission for each patient was classified into one of nine distinct disease categories: cardiovascular, respiratory, gastrointestinal, neurologic, metabolic/endocrine, hematologic, genitourinary, musculoskeletal/skin and trauma. These admission diagnoses were determined by the ICU specialist on duty and subsequently verified by an intensivist. Other information that were collected were patient's mechanical ventilation status and the availability of Glasgow Coma Scale (GCS) score.

The physiological variables that were collected were heart rate, mean blood pressure, temperature, respiratory rate, hematocrit, white blood cell count, creatinine, urine output, blood urea nitrogen, sodium, albumin, bilirubin, glucose, PaO_2_, acid-base abnormalities and Glasgow Coma Scale (GCS) score. Most of the physiological variables that were easily available were monitored on an hourly basis. However, variables that required laboratory evaluations were collected approximately twice per day. The APACHE IV severity of illness scores were assigned to the physiological variables, where the Acute Physiology Score (APS) [3] was computed for each patient. Calculation of APS was manually performed using Microsoft^®^ Excel (2007), by combining the scores for all of the worst physiological variables within the first day of ICU stay for each patient. An imputation method was applied for patients with missing laboratory data, where these missing observations were assumed normal and were substituted with midpoint values that were defined in APACHE IV. Patients with incomplete first day APS information were excluded from analysis so as not to affect model accuracy.

### Risk factors

Univariable analysis was performed on all candidate variables using Bayesian MCMC approach in order to identify significant main risk factors. Admissions between 1 January 2009 and 31 December 2009 were used in the construction of the univariate models. Univariate logistic regression models were fitted for each of the candidate variables, with a binary outcome of "1" indicating death in ICU and "0" for being alive upon discharge from ICU. Independent variables were categorized into continuous and categorical variables. The continuous variables included age, APS and pre-ICU length of stay, whereas the other variables were categorical in nature.

Model development and inference were performed using WinBUGS[15],which is a software that applies Gibbs sampling approach in estimation of model parameters. Model specification in WinBUGS required specification of a likelihood for the outcome variable, a *logit* expression in the form of a linear combination of risk factor(s), prior distributions and initial values for the regression parameters and input data. Non-informative priors were used in the development of models in this study due to lack of information on the regression parameters. A weakly informative Gaussian prior distribution with zero mean and a fixed large variance (σ^2^ = 1000) were assigned to the regression parameters in the univariate models. Three multiple parallel chains with different starting points were applied in all simulation work in order to monitor convergence of the chains. The univariate models were updated by running the multiple chains for 500,000 iterations each, where the initial 100,000 burn-in samples were discarded from analysis. Model convergence was monitored in WinBUGS through the estimated Monte Carlo errors for the posterior means, trace plots and Brooks-Gelman-Rubin (BGR) diagnostic.

The significance of the estimated regression coefficients for each variable was tested following two criteria; by using likelihood ratio test and Deviance Information Criterion (DIC)[16], and by looking at the credible intervals for the posterior means of each variable. The likelihood ratio test involved comparison of the -2 log likelihoods of the model with a variable of interest versus the model without the variable of interest. For each univariate model, a variable was considered as significant if the *p*-value for the likelihood ratio test was less than 0.25 and if the 75% credible intervals did not contain the value zero. The threshold of 0.25 was chosen based on the argument that traditional *p*-values of 0.05 or 0.10 were often ineffective in screening important variables at the univariate level[17]. Variables that satisfied both criteria were then fitted into four different combinations of multivariate logistic regression models for further evaluation.

### Multivariable Models and Performance Measures

Development of the multivariable models involved data from 916 admissions between January 1, 2009 and December 31, 2009. A total of 195 admissions between January 1, 2010 and June 30, 2010 were used for model validation. All variables that satisfied the screening criteria at the univariate level were fitted into several combinations of multivariable models. The variables were collectively tested for their significance and possible interactions between variables were evaluated. Linearity assumption for the continuous variables was assessed through LOESS (Locally Weighted Scatterplot Smoothing) plots [18] and non-linear transformation tests [19].

A weakly informative Gaussian prior distribution with zero mean and a fixed large variance (σ^2^ = 1000) was applied to the regression parameters in the multivariable models. Simulation runs for three parallel chains were fixed at one million iterations, with the first 100,000 samples discarded in the burn-in period to eliminate the effect of initial values. Chain convergence was monitored through trace and autocorrelation plots, Brooks-Gelman-Rubin (BGR) diagnostic and the estimated Monte Carlo errors for the posterior means.

The overall predictive performance of the models was assessed through the Standardized Mortality Ratio (SMR) and Brier score [20]. The SMR values and their corresponding 95% confidence intervals were calculated for each model. The SMR was computed as the ratio of mean of observed deaths in ICU over the mean of predicted deaths in the ICU, in which a ratio of 1.0 indicated that the overall expected and observed death rates in the ICU were the same. The Brier scores for each model were computed by taking into account the squared differences between observed and predicted outcomes [21]. The decision space for a useful model was restricted to (0, 0.25), where a model with a smaller Brier score was considered to have better accuracy [22].

Model discrimination was measured through area under receiver operating characteristic curves (AUC) using a non-parametric approach [23]. An AUC of 1.0 implied perfect discrimination, where all predicted outcomes were the same as the observed outcomes for all patients. In this study, discrimination was considered good if AUC> 0.8. Analysis of AUC was performed using MedCalc 10.4 (Medcalc Software, Mariakerke, Belgium). Model calibration was evaluated through the Hosmer-Lemeshow goodness-of-fit test [24] and calibration curves. The Hosmer-Lemeshow goodness-of-fit test measured the overall model calibration by comparing observed and predicted probabilities of death for different subgroups of patients. A model was considered well-calibrated if the *p*-value for this test was greater than 0.05. Calibration curves were plotted to compare differences in observed and predicted in-ICU mortality rates across ten equal-sized groups.

A model's overall goodness-of-fit was measured through Deviance Information Criterion (DIC), which was estimated from samples that were generated by Bayesian MCMC simulation. The DIC allowed comparison of several candidate models, in which a model with a lower DIC value was considered to have an overall better fit. This criterion was used in selecting the best model for HSA ICU in our study. For comparison purpose, the estimates and standard errors of regression coefficients in the multivariable models were also obtained through the maximum likelihood method using S-PLUS version 8.1 (Insightful Corporation). The performances of the frequentist models were then compared against the Bayesian models.

## Results

### Patient Characteristics

Table 1 shows the comparison in patient characteristics for admissions to HSA ICU between the developmental and validation data sets. These statistics revealed almost similar patient profiles in the two data sets and no temporal changes in the baseline characteristics of patients. Male patients accounted for almost 60% of the total admissions. Patients were categorized into four major ethnic groups (Malay, Chinese, Indian and Others) according to the population in Malaysia. Those who did not belong to any of these three main ethnic groups were classified in a category named Others. Malay patients formed the majority, with more than 50% of the total admissions. This was followed by Chinese (24.7%), Indian (10.8%) and Others (8.6%). More than 80% of patients required mechanical ventilation. Approximately one-quarter of the total admissions had no Glasgow Coma Scale (GCS) score on the first day of ICU admission as these patients were either sedated or paralyzed.

**Table 1:** Comparison of casemix for admissions to HSA ICU between developmental and validation datasets.

Patient characteristics	Stage 1^#^ (Developmental data set)	Stage 2* (Validation data set)	Overall
Total patients	916	195	1,111
Age (mean ± SD, in years)	43.4 ±17.6	43.6 ± 18.5	43.5 ± 17.7
Acute Physiology Score, APS (mean ± SD)	69.6 ± 31.9	63.3 ± 33.1	68.5 ± 32.2
Male (%)	60.6	61.5	60.8
Ethnicity (%)			
Malay	56.4	53.3	55.9
Chinese	24.1	27.2	24.7
Indian	10.7	11.3	10.8
Others	8.7	8.2	8.6
ICU admission source (%)			
Floor	47.3	49.7	47.7
Other special care unit	12.2	10.8	12.0
Operating room	40.5	39.5	40.3
Emergency surgery (%)	36.6	35.4	36.4
Pre ICU length of stay (mean ± SD, in days)	1.1 ± 2.3	0.8 ± 1.7	1.1 ± 2.2
Mechanically ventilated (%)	83.0	86.7	83.6
Unable to obtain Glasgow Coma Scale (GCS) score (%)	23.1	35.9	25.4
Dead in ICU (%)	18.8	16.9	18.5
With at least one co-morbidities (%)	3.7	4.1	3.8
Diabetes (%)	20.1	21.5	20.3
Disease categories (%)			
Trauma	20.6	19.0	20.3
Cardiovascular	22.3	19.0	21.7
Respiratory	18.2	20.5	18.6
Neurologic	17.1	16.4	17.0
Gastrointestinal	11.1	9.2	10.8
Genitourinary	7.1	9.2	7.5
Metabolic/endocrine	2.6	1.5	2.4
Musculoskeletal/skin	0.5	1.5	0.7
Hematologic	0.3	3.6	0.9

ICU: intensive care unit; SD: standard deviation

^#^ Data collected from 1 January 2009 to 31 December 2009

* Data collected from 1 January 2010 to 30 June 2010

Patients were mostly admitted from the ward/recovery room (48%) and operating room/emergency room (40%). Non-operative and post-operative admissions were almost equally distributed. However, the percentage of emergency surgery patients was much higher compared to the percentage of elective surgeries. The majority of post-operative admissions were due to trauma, with a high percentage coming from road accident patients who were transferred from the Accident and Emergency unit. On the other hand, cardiovascular and respiratory diseases were the main causes of ICU admission for the non-operative admissions. The ICU recorded low number of admissions for patients with musculoskeletal/skin and hematologic diseases.

Admissions to HSA ICU throughout the period of study generally consisted of a younger set of patients, with an overall mean age of 43.5 years (±17.7 years). The majority of younger patients (below age 30 years old) were admitted due to trauma-related illnesses, whereas patients between 30 to 50 years old were mostly admitted because of cardiovascular and neurologic diseases. A large percentage of patients in their 50s and 60s were admitted due to cardiovascular and respiratory ailments, while older patients (> 70 years) were mostly admitted because of gastrointestinal problems. The cohort of patients had a low co-morbidity load, where less than 5% reported that they had at least one chronic health disease. However, approximately 20% of patients revealed that they had diabetes, where the prevalence of diabetes was higher in older age groups (> 50 years). There was no difference in the prevalence of diabetes among the three major ethnic groups (Malay, Chinese and Indian). The total number of in-ICU deaths throughout the period of study was 205 (18.5%). HSA ICU patients generally exhibited greater degree of severity of illness with higher APS values. The overall mean of first day APS for admissions to HSA ICU throughout the period of study was observed to be high at 68.5.

An analysis of the monthly ICU admission rates in year 2009 revealed lower number of admissions in the first two months (January and February). A peak in admissions was observed between August to December. The majority of admissions in August were due to respiratory diseases. This period coincided with the national flu pandemic that involved the A(H1N1) virus. Trauma patients formed a high percentage of admissions from October to December. The increase in trauma admissions was mostly due to road accident patients who were transferred from the Accident and Emergency (A&E) unit. Despite the variations in monthly admission rates, there were no seasonal patterns in ICU mortality as fluctuations in the percentage of deaths in ICU were not significant across all months. These results indicated that there was no significant association between the frequency of ICU admissions and mortality outcomes in this study.

### Statistical modeling and analyses

Table 2 shows the results of univariable analyses for all potential risk factors. Variables that were found to be significant at the univariate level were gender (OR:0.53), APS (OR:1.03), mechanical ventilation status (OR:13.2), absence of GCS score (OR:2.25), presence of chronic health (OR:1.65) and diabetes (OR:1.71). Other variables such as age, ethnicity, ICU admission source, emergency surgery and pre ICU length of stay were found to be not statistically significant based on their 75% credible intervals and likelihood ratio tests.

**Table 2:** Univariate analysis for potential risk factors.

Variable	Posterior mean	75% Credible Interval	SE	MC error	Odds ratio (OR)
Age	0.0049	(-0.001, 0.011)	0.0002	1.59E-05	1.00
APS	0.0332	(0.029, 0.037)	0.0001	3.00E-05	1.03
Ethnicity					
Chinese	0.1313	(-0.116, 0.379)	0.0071	2.89E-04	1.14
Indian	-0.0921	(-0.449, 0.265)	0.0102	3.33E-04	0.91
Others	0.0065	(-0.374, 0.387)	0.0109	3.49E-04	1.01
ICU admission source					
Another special care unit	-0.0417	(-0.374, 0.291)	0.0095	3.33E-04	0.96
Operating room	-0.0594	(-0.281, 0.162)	0.0064	2.76E-04	0.94
Gender	-0.6305	(-0.856, -0.405)	0.0065	5.01E-04	0.53
Presence of chronic health (yes)	0.5016	(0.279, 0.724)	0.0064	4.10E-04	1.65
Absence of GCS score	0.8117	(0.583, 1.040)	0.0066	7.10E-04	2.25
Emergency surgery	0.1299	(-0.081, 0.341)	0.0061	2.61E-04	1.14
Mechanically ventilated (yes)	2.58	(1.938, 3.222)	0.0185	0.004016	13.20
Pre ICU length of stay (square root, in days)	-0.0056	(-0.154, 0.143)	0.0043	2.06E-04	0.99
Diabetes (yes)	0.5389	(0.296, 0.781)	0.0070	5.04E-04	1.71
Chronic health groups					
AIDS	1.593	(-0.581, 3.767)	0.0624	0.002232	4.92
Cancer	-25.84	(-47.587, -4.094)	0.6248	0.01566	<0.01
Cirrhosis	0.7163	(-0.465, 1.897)	0.0339	0.001282	2.05
Hepatic failure	-24.2	(-46.568, -1.833)	0.6426	0.01615	<0.01
Immunosuppression	-0.4463	(-1.481, 0.589)	0.0297	7.56E-04	0.64
Leukemia/multiple myeloma	2.626	(0.803, 4.449)	0.0524	0.002437	13.82
Lymphoma	1.591	(-0.585, 3.767)	0.0625	0.002294	4.91
Disease groups					
Cardiovascular	0.0777	(-0.217, 0.373)	0.0085	4.97E-04	1.08
Gastrointestinal	-0.0182	(-0.382, 0.345)	0.0104	5.25E-04	0.98
Genitourinary	-2.56	(-3.532, -1.588)	0.0279	0.001402	0.08
Hematologic	2.359	(0.533, 4.185)	0.0525	0.002369	10.58
Metabolic/endocrine	-0.182	(-0.856, 0.492)	0.0194	6.65E-04	0.83
Musculoskeletal/skin	-25.75	(-47.531, -3.969)	0.6258	0.01579	<0.01
Neurologic	-0.9518	(-1.332, -0.571)	0.0109	8.49E-04	0.39
Respiratory	-0.2765	(-0.602, 0.0491)	0.0094	5.46E-04	0.76

SE: standard error; MC: Monte Carlo; GCS: Glasgow Coma Scale

Note: p-values for likelihood ratio tests for all variables were < 0.25.

The following combinations of logistic regression models with their respective variables were then proposed:

M1: age, gender, APS, mechanical ventilation, absence of GCS score, admission diagnoses, presence of chronic health.

M2: age, gender, APS, mechanical ventilation, absence of GCS score, admission diagnoses, diabetes.

M3: age, gender, APS, mechanical ventilation, absence of GCS score, diabetes, trauma, interaction (APS x trauma).

M4: age, gender, mechanical ventilation, absence of GCS score, admission diagnoses, presence of chronic health, worst physiological variables in ICU Day 1 (normal/abnormal).

Due to the extremely low percentage of patients with existing co-morbidities, presence of chronic health (yes/no) was used as a variable in model M1, instead of the seven chronic health categories defined in APACHE IV. The high prevalence of diabetes in HSA ICU patients and in Malaysia [25–26] suggested the potential of diabetes as a risk factor. In order to investigate the effect of diabetes on mortality risk, this variable was included in model M2 in place of presence of chronic health. In models M1 and M2, patients were grouped into one of nine individual admission diagnoses. Trauma was chosen as the reference category for admission diagnoses due to the large percentage of patients in this group. In model M3, we tried to simplify classification of admission diagnoses by re-classifying patients into two groups, i.e. trauma and non-trauma.

The APS was considered an important predictor of in-ICU deaths in this study, and was included in models M1–M3. We explored an alternative approach in assessing the degree of severity of illness in ICU patients, without involving the use of APS. Variables in model M1 were entered into model M4, except for APS. Instead, the worst values for each physiological variable in ICU Day 1 were dichotomously coded as normal/abnormal and were included in model M4. Classification of abnormality was based on definitions in APACHE IV, where missing values were assumed normal.

Table 3 shows the results of the univariate tests for each of the abnormal worst physiological variables. Three variables (abnormal mean blood pressure, abnormal total respiratory rate and abnormal hematocrit) were not statistically significant based on their 75% credible intervals and were not entered into the multivariable models. The rest of the abnormal physiological variables were collectively assessed for their statistical significance at the multivariate level, where those that were significant were finally included in model M4.

**Table 3:** Univariate analysis for physiological variables.

Physiological variable	Posterior mean	75% Credible Interval	SE	Odds ratio (OR)	Significant
Abnormal heart rate	1.504	(1.139, 1.869)	0.010	4.50	Yes
Abnormal mean blood pressure	0.2459	(-0.454, 0.946)	0.020	1.28	No
Abnormal temperature	1.1	(0.862, 1.338)	0.007	3.00	Yes
Abnormal total respiratory rate	-0.08762	(-0.295, 0.119)	0.006	0.92	No
Abnormal hematocrit	-0.2418	(-0.580, 0.096)	0.010	0.79	No
Abnormal white blood cell count	0.7379	(0.512, 0.964)	0.007	2.09	Yes
Abnormal creatinine	0.5832	(0.373, 0.793)	0.006	1.79	Yes
Abnormal total urine output	0.3432	(0.085, 0.601)	0.007	1.41	Yes
Abnormal blood urea nitrogen	1.123	(0.888, 1.358)	0.007	3.07	Yes
Abnormal sodium	0.9296	(0.692, 1.167)	0.007	2.53	Yes
Abnormal albumin	1.301	(1.078, 1.524)	0.006	3.67	Yes
Abnormal bilirubin	0.9463	(0.657, 1.235)	0.008	2.58	Yes
Abnormal glucose	0.7914	(0.579, 1.004)	0.006	2.21	Yes
Abnormal PaO_2_	1.551	(1.187, 1.915)	0.010	4.72	Yes
Abnormal ph-PaCO_2_ relationship	1.668	(1.261, 2.075)	0.012	5.30	Yes

SE: standard error

Note: p-values for likelihood ratio tests for all physiological variables were < 0.25.

### Performance and validation results of proposed models

The MCMC diagnostics for all four models revealed no specific trends or irregularities in the trace and density plots. The Brooks-Gelman-Rubin (BGR) plots also indicated that convergence was achieved in the multiple parallel chain simulations for all models. The estimated coefficients and odds ratios for variables in models M1 and M2 are presented in Tables 4 and 5 respectively. The regression coefficients and standard errors obtained through maximum likelihood estimation (MLE) method are also shown in the two tables. The Bayesian and MLE estimates were concordant for most of the variables in models M1 and M2. However, the standard errors obtained through the Bayesian approach were consistently much smaller compared to the frequentist (MLE) standard errors for all variables in both models. Differences in some of the admission diagnoses were due to small sample sizes in these disease categories.

**Table 4:** Estimated regression coefficients and odds ratios for variables in model M1.

Variable	Bayesian estimation			Frequentist (MLE)
	Posterior Mean (95% CI)	SE	Odds ratio (95% CI)	Coefficient ± SE
Age	-0.004 (-0.019, 0.010)	0.0002	1.00 (0.98, 1.01)	-0.004 ± 0.007
Gender (female)	-0.649 (-1.142, -0.172)	0.008	0.52 (0.32, 0.84)	-0.582 ± 0.224
APS	0.044 (0.034, 0.054)	0.0002	1.04 (1.03, 1.05)	0.039 ± 0.004
No GCS*	1.756 (1.204, 2.337)	0.01	5.79 (3.33, 10.35)	1.589 ± 0.254
Mechanical ventilation	0.827 (-0.344, 2.200)	0.021	2.29 (0.71, 9.03)	0.688 ± 0.584
With chronic health	0.324 (-0.197, 0.846)	0.009	1.38 (0.82, 2.33)	0.295 ± 0.241
Admission diagnoses				
Cardiovascular	0.003 (-0.680, 0.688)	0.012	1.00 (0.51, 1.99)	-0.021 ± 0.317
Respiratory	-0.27 (-0.971, 0.423)	0.012	0.76 (0.38, 1.53)	-0.271 ± 0.325
Gastrointestinal	-0.216 (-1.035, 0.590)	0.014	0.81 (0.36, 1.80)	-0.214 ± 0.376
Neurologic	-0.602 (-1.361, 0.131)	0.013	0.55 (0.26, 1.14)	-0.559 ± 0.347
Metabolic/endocrine	-0.291 (-1.663, 0.982)	0.022	0.75 (0.19, 2.67)	-0.231 ± 0.600
Hematologic	3.251 (0.052, 7.059)	0.058	25.82 (1.05, 1163.28)	2.637 ± 1.397
Genitourinary	-1.946 (-3.948, -0.380)	0.03	0.14 (0.02, 0.68)	-1.651 ± 0.785
Musculoskeletal/skin	-26.47 (-71.420, -2.571)	0.619	<0.01 (0.00, 0.08)	-6.172 ± 6.438

CI: credible interval; GCS: Glasgow Coma Scale; MLE: maximum likelihood estimation; SE: standard error

* NoGCS refers to patients who had no Glasgow Coma Scale (GCS) scores either due to sedation or paralysis.

**Table 5:** Estimated regression coefficients and odds ratios for variables in model M2.

Variable	Bayesian estimation			Frequentist (MLE)
	Posterior Mean (95% CI)	SE	Odds ratio (95% CI)	Coefficient ± SE
Age	-0.005 (-0.019, 0.010)	0.0002	1.00 (0.98, 1.01)	-0.004 ± 0.007
Gender (female)	-0.644 (-1.137, -0.166)	0.008	0.53 (0.32, 0.85)	-0.577 ± 0.224
APS	0.044 (0.034, 0.054)	0.0002	1.04 (1.04, 1.06)	0.039 ± 0.004
No GCS*	1.761 (1.208, 2.341)	0.01	5.82 (3.35, 10.39)	1.591 ± 0.254
Mechanical ventilation	0.834 (-0.343, 2.216)	0.021	2.30 (0.71, 9.17)	0.691 ± 0.584
Diabetes	0.360 (-0.187, 0.910)	0.009	1.43 (0.83, 2.49)	0.323 ± 0.253
Admission diagnoses				
Cardiovascular	0.007 (-0.678, 0.696)	0.012	1.01 (0.51, 2.01)	-0.016 ± 0.317
Respiratory	-0.250 (-0.945, 0.441)	0.012	0.78 (0.39, 1.55)	-0.253 ± 0.322
Gastrointestinal	-0.208 (-1.027, 0.600)	0.014	0.81 (0.36, 1.82)	-0.206 ± 0.376
Neurologic	-0.576 (-1.334, 0.156)	0.013	0.56 (0.26, 1.17)	-0.534 ± 0.346
Metabolic/endocrine	-0.303 (-1.679, 0.974)	0.022	0.74 (0.19, 2.65)	-0.241 ± 0.601
Hematologic	3.586 (0.405, 7.378)	0.058	36.09 (1.50, 1600.39)	2.939 ± 1.385
Genitourinary	-1.92 (-3.919, -0.356)	0.03	0.15 (0.02, 0.70)	-1.626 ± 0.784
Musculoskeletal/skin	-26.53 (-71.550, -2.609)	0.62	<0.01 (0.00, 0.07)	-6.187 ± 6.430

CI: credible interval; GCS: Glasgow Coma Scale;MLE: maximum likelihood estimation; SE: standard error

* NoGCS refers to patients who had no Glasgow Coma Scale (GCS) scores either due to sedation or paralysis.

In both models, age had negligible effect on mortality risk, while the odds of dying for female patients was 50% lower compared to male patients. Increasing APS and absence of GCS score were associated with a higher mortality risk. For every increase of one point in APS, the odds of dying increased by 4%. Presence of chronic health/diabetes and being mechanically ventilated were not significant in models M1 and M2, although they were significant at the univariate level. The LOESS plots and non-linearity transformation test did not suggest violations in the linearity assumptions for age and APS variables in models M1 and M2. Interaction terms between variables in these two models were also not statistically significant.

The estimated coefficients and odds ratios for variables in model M3 are shown in Table 6. Gender, APS and absence of GCS score were significant based on the 95% credible intervals of the posterior means. Mechanical ventilation and diabetes were not significant in this model. Trauma patients had lower odds of dying (OR: 0.19) compared to patients from other admission diagnoses. Positive interaction was detected between APS and trauma, with the interaction term being significant at a 5% level of significance. Other interactions between trauma and gender, as well as, trauma and absence of GCS score were also tested. However, these interaction terms were not statistically significant and were omitted in model M3.

**Table 6:** Estimated regression coefficients and odds ratios for variables in model M3.

Variable	Bayesian estimation			Frequentist (MLE)
	Posterior Mean (95% CI)	SE	Odds ratio (95% CI)	Coefficient ± SE
Age	-0.002 (-0.016, 0.012)	0.0002	1.00 (0.98, 1.01)	-0.002 ± 0.007
Gender (female)	-0.609 (-1.080, -0.155)	0.008	0.54 (0.34, 0.86)	-0.554 ± 0.216
APS	0.039 (0.030, 0.049)	0.0002	1.04 (1.03, 1.05)	0.036 ± 0.004
No GCS	1.902 (1.360, 2.476)	0.009	6.70 (3.90, 11.89)	1.744 ± 0.249
Mechanical ventilation	0.702 (-0.396, 1.987)	0.02	2.02 (0.67, 7.29)	0.597 ± 0.558
Diabetes	0.354 (-0.170, 0.879)	0.009	1.42 (0.84, 2.41)	0.321 ± 0.244
Trauma	-1.656 (-3.309, -0.132)	0.027	0.19 (0.04, 0.88)	-1.451 ± 0.744
APS x trauma	0.025 (0.007, 0.044)	0.0003	1.03 (1.01, 1.05)	0.022 ± 0.009

CI: credible interval; MLE: maximum likelihood estimation; SE: standard error

The physiological variables that met inclusion criteria in model M4 were abnormal heart rate, abnormal temperature, abnormal white blood cell count, abnormal blood urea nitrogen, abnormal sodium, abnormal albumin, abnormal bilirubin and abnormal ph-PaCO_2_ relationship (Table 7). Gender and mechanical ventilation were statistically significant, whereas age, absence of GCS score and presence of chronic health were found to be not significant in model M4. There were no substantial differences in the point estimates obtained through Bayesian and frequentist methods in both models M3 and M4. Similar to the first two models, the Bayesian approach produced smaller standard errors compared to the frequentist method.

**Table 7:** Estimated regression coefficients and odds ratios for variables in model M4.

Variable	Bayesian estimation			Frequentist (MLE)
	Posterior Mean (95% CI)	SE	Odds ratio (95% CI)	Coefficient± SE
Age	-0.001 (-0.016, 0.014)	0.0002	1.00 (0.98, 1.01)	-0.001 ± 0.007
Gender (female)	-0.551 (-1.046, -0.067)	0.0082	0.58 (0.35, 0.93)	-0.479 ± 0.222
No GCS	0.391 (-0.080, 0.864)	0.0079	1.48 (0.92, 2.37)	0.331 ± 0.212
Mechanical ventilation	2.289 (1.148, 3.665)	0.0212	9.87 (3.15, 39.06)	1.959 ± 0.563
With chronic health	0.500 (-0.039, 1.042)	0.0091	1.65 (0.96, 2.83)	0.438 ± 0.244
Admission diagnoses				
Cardiovascular	-0.241 (-0.987, 0.502)	0.0125	0.79 (0.37, 1.65)	-0.221 ± 0.337
Respiratory	-0.234 (-0.964, 0.486)	0.0122	0.79 (0.38, 1.63)	-0.204 ± 0.329
Gastrointestinal	-0.492 (-1.337, 0.339)	0.0141	0.61 (0.26, 1.40)	-0.430 ± 0.379
Neurologic	-0.46 (-1.260, 0.319)	0.0133	0.63 (0.28, 1.38)	-0.409 ± 0.361
Metabolic/endocrine	-0.375 (-1.797, 0.949)	0.0231	0.69 (0.17, 2.58)	-0.292 ± 0.607
Hematologic	2.379 (-0.957, 6.183)	0.0595	10.79 (0.38, 484.44)	1.869 ± 1.411
Genitourinary	-1.91 (-3.926, -0.325)	0.0303	0.15 (0.02, 0.72)	-1.553 ± 0.781
Musculoskeletal/skin	-26.27 (-71.460, -2.202)	0.6215	<0.01 (0.00, 0.11)	-5.779 ± 6.407
Abnormal physiological^†^				
Heart rate	0.943 (0.252, 1.688)	0.0121	2.57 (1.29, 5.41)	0.834 ± 0.331
Temperature	0.893 (0.408, 1.384)	0.0082	2.44 (1.50, 3.99)	0.786 ± 0.219
White blood cell count	0.614 (0.128, 1.102)	0.0082	1.85 (1.14, 3.01)	0.553 ± 0.219
Blood urea nitrogen	0.665 (0.142, 1.203)	0.0089	1.94 (1.15, 3.33)	0.575 ± 0.241
Sodium	0.719 (0.226, 1.216)	0.0083	2.05 (1.25, 3.37)	0.622 ± 0.222
Albumin	0.912 (0.453, 1.380)	0.0078	2.49 (1.57, 3.97)	0.793 ± 0.207
Bilirubin	0.84 (0.217, 1.465)	0.0105	2.32 (1.24, 4.33)	0.752 ± 0.279
pH-PaCO_2_ relationship	0.974 (0.210, 1.808)	0.0135	2.65 (1.23, 6.10)	0.843 ± 0.366

CI: credible interval; MLE: maximum likelihood estimation; SE: standard error

^†^ Abnormal physiological variables that were found to be significant in multivariable model.

The performances and validation results of the four models are presented in Table 8. As expected, the performances of the Bayesian models were comparable to the frequentist models, especially in AUC and Brier Scores. The results from the Hosmer-Lemeshow goodness-of-fit test suggested that the frequentist method produced slightly better calibration across different subgroups of patients in all four models. However, the SMR values for the Bayesian models were much better than their counterpart frequentist models. Moreover, the deviance values in all four Bayesian models were significantly lower than the frequentist models. This implied that better model fit was achieved through the Bayesian method.

**Table 8:** Performances of all four models based on validation data set.

Model	DIC	Deviance	AUC (95% CI)	Brier score	H-L statistic (*p*)	SMR (95% CI)
Bayesian						
M1	695.4	635.7	0.809 (0.747, 0.862)	0.113	9.06 (*p* = 0.3373)	0.909 (0.626, 1.277)
M2	695.0	634.8	0.802 (0.739, 0.855)	0.118	8.53 (*p* = 0.3835)	0.862 (0.593, 1.211)
M3	696.0	644.4	0.792 (0.728, 0.846)	0.113	18.06 (*p* = 0.0208)	0.952 (0.655, 1.337)
M4	719.4	643.4	0.807 (0.744, 0.860)	0.117	12.37 (*p* = 0.1354)	1.013 (0.697, 1.423)
Frequentist						
M1	-	670.56	0.810 (0.748, 0.862)	0.113	3.97 (*p* = 0.8598)	0.858 (0.590, 1.205)
M2	-	670.43	0.804 (0.741, 0.857)	0.117	8.47 (*p* = 0.389)	0.817 (0.562, 1.147)
M3	-	680.63	0.792 (0.729, 0.847)	0.114	14.84 (*p* = 0.0623)	0.882 (0.607, 1.238)
M4	-	684.77	0.806 (0.744, 0.859)	0.116	8.92 (*p* = 0.3491)	0.931 (0.641, 1.307)

DIC: deviance information criterion; AUC: area under receiver operating characteristic curve; CI: confidence interval; H-L statistic: Hosmer-Lemeshow statistic; SMR: standardized mortality ratio

**Fig 1:**
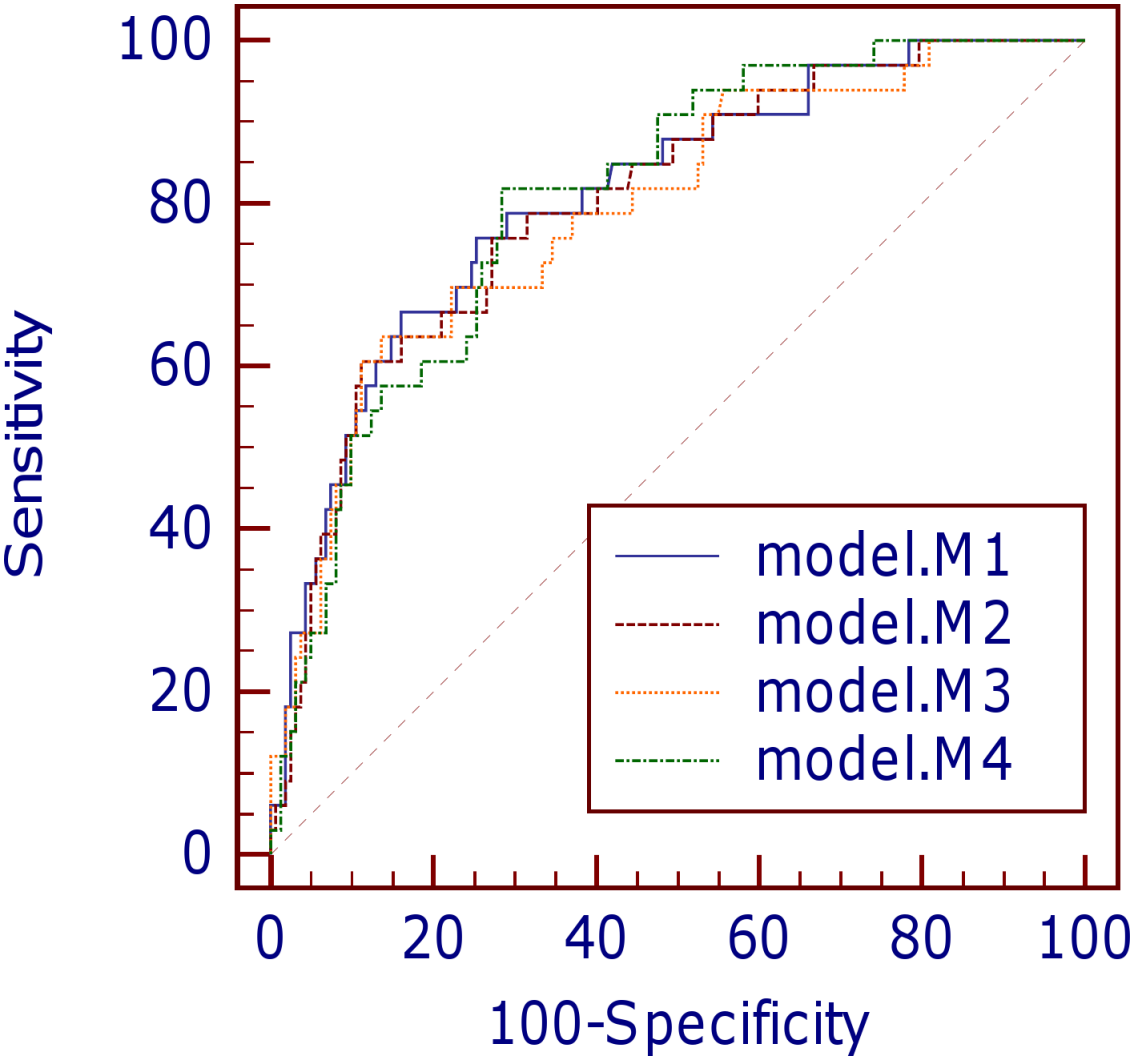
Comparison of receiver operating characteristic curves for four predictive models.

**Fig 2:**
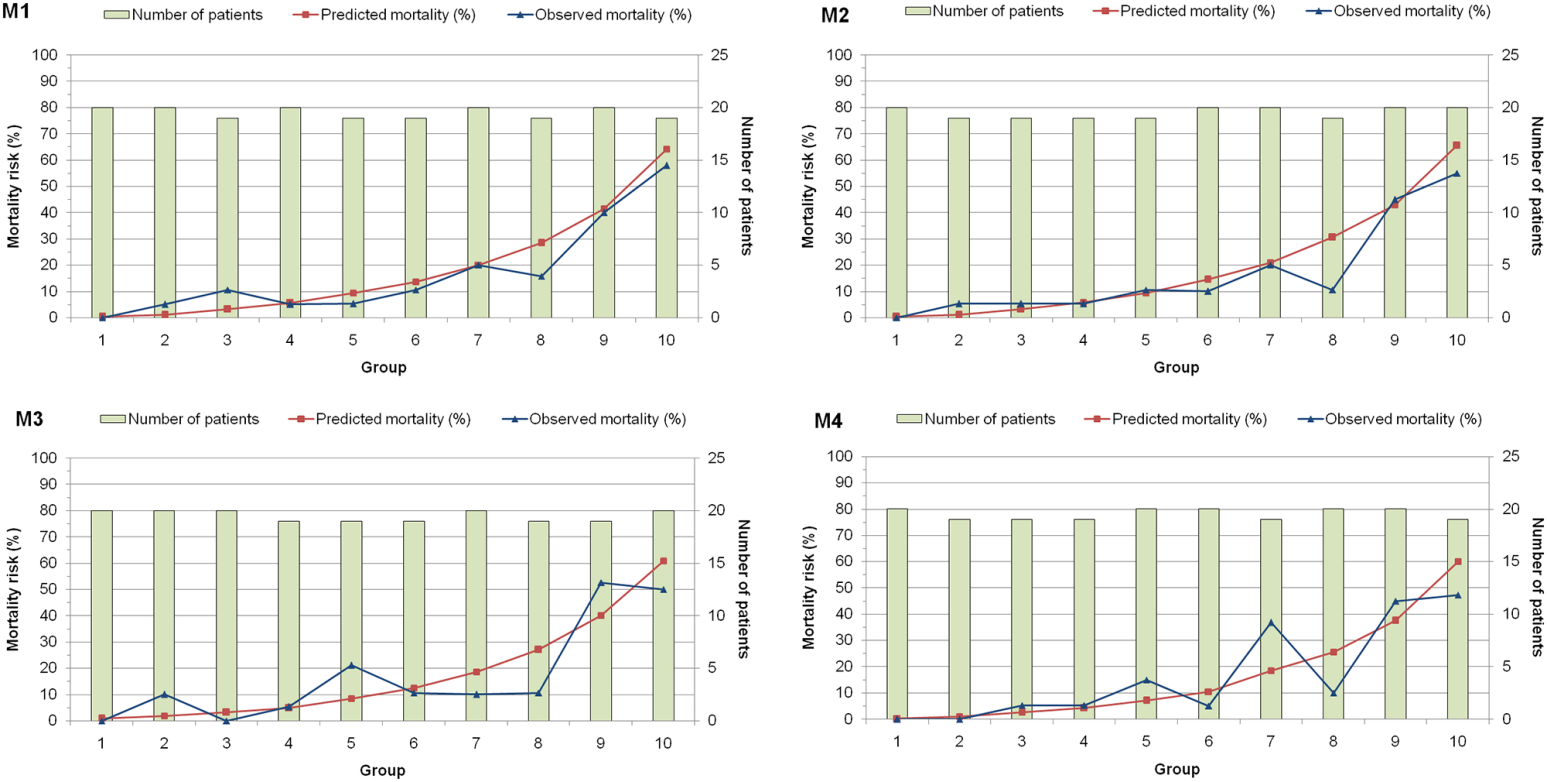
Calibration curves of the four models.

The small Brier scores (0.113–0.118) in the four models indicated good overall accuracy. All the Bayesian models exhibited good discrimination power, with marginal differences in AUC values (Fig 1 and Table 8). Discrimination in models M1, M2 and M4 were equally good (AUC > 0.8), whereas model M3 had the lowest AUC among the four models. The Hosmer-Lemeshow goodness-of-fit tests indicated that calibration was good for all the Bayesian models except model M3 (all *p*-values > 0.05 except for model M3) (Table 8). Further inspection of the calibration curves (Fig 2) revealed close agreement between observed and predicted values across ten equal-sized groups in models M1 and M2. However, slight discrepancies between observed and predicted mortality rates were observed in certain patient groups in models M3 and M4. On the whole, models M1, M2 and M3 overestimated in-ICU mortality, with SMR < 1.0. There was no significant difference between the overall mean predicted and mean observed mortality in model M4 (SMR = 1.013).

There were no significant differences in DIC for models M1, M2 and M3. Nevertheless, the DIC in model M4 was distinctly higher compared to the other models. Although the higher DIC value could probably be affected by the higher number of explanatory variables in model M4, this large difference of more than 20 units implied strong evidence against model M4 compared to the other models.

## Discussion

This study has shown that Bayesian MCMC approach can be successfully applied as an alternative in developing ICU prognostic models, where the four proposed models were capable of predicting mortality risk in HSA ICU. Variables such as gender, APS, inability to assess GCS score and being mechanically ventilated were found to be important determinants of HSA ICU mortality risk. Female patients had lower risks of dying in ICU compared to male patients. Despite being a multiracial country, ethnicity was not a significant predictor of death in the cohort of HSA ICU patients. One possible explanation for this could be that Malaysians generally have similar dietary and eating habits although they come from culturally diverse backgrounds. Moreover, the increasing number of inter-ethnic marriages over the years could have also contributed towards integration of cultural values and lifestyles in the Malaysian society.

Patient characteristics in the Malaysian ICU were generally different from ICUs in the western countries. For instance, the mean age reported in APACHE IV was in the range of 60s, whereas the average age of patients admitted to HSA ICU was in the 40s. Young patients formed the majority of admissions in HSA ICU, with approximately 30% being less than 30 years of age. In this study, age was found to have no effect modification when assessed across other variables. We observed no significant correlation between age and probability of mortality. Our study also revealed no positive association between age and APS. We found that patients who died were not necessarily older with higher APS, as there were quite a number of younger patients with high APS values. The group of younger patients mostly suffered from severe trauma injuries, predominantly caused by motorcycle road accidents. The high percentage of patients being admitted for motorcycle road accidents corroborated the national statistics of road injury and fatality involving motorcyclists in Malaysia [27]. These patients also contributed towards a higher number of emergency surgeries in the ICU.

APS remained a relevant and important risk factor in this study, where increasing APS was found to be positively associated with in-ICU mortality risk in models M1–M3. There was no significant improvement in risk prediction when presence of chronic health variable in model M1 was replaced with diabetes in model M2. Despite the high percentage of diabetic patients, diabetes was not statistically significant when combined with other variables in model M2. This finding supported the theory that although diabetic patients were susceptible to more complications, diabetes was not associated with increased in-ICU mortality risk [28–30].

Models M1 and M2 generally performed well, with good discrimination and calibration power. The prediction accuracy in Model M1 was considered marginally better than model M2, in SMR and Brier score measures. The performance of model M3 was found to be lacking in calibration compared to models M1 and M2. The main difference between model M3 and models M1/M2 was in the classification of ICU admission diagnoses. Despite being a simpler model, calibration in model M3 was found to be inadequate across the groups of patients with different risk profiles. This finding favored the option of retaining the original classification of ICU admission diagnoses as in models M1 and M2, over the simplified classification of trauma/non-trauma in model M3. On the other hand, model M4 was considered to have the poorest model fit since it had the worst DIC among the four models. These findings supported model M1 as the preferred choice in this study, with the best overall fit, discrimination and calibration power.

In this study, both Bayesian and frequentist (MLE) methods produced results that were close in agreement and similar conclusions in terms of model performance. There were no substantial differences between the estimates obtained through these two methods. This was probably due to the data set being sufficiently large, especially for the MLE approach. In addition, a large number of iterations was also employed in the Bayesian MCMC simulations in order to achieve model convergence. The advantage of the Bayesian method lies in it being a data-driven approach that allows the data to speak for themselves. The ability to quantify uncertainty in the parameters offered more flexibility in the Bayesian modeling approach. Although absence of prior experience necessitated the use of non-informative (vague) prior distributions, the Bayesian approach was able to provide results that were consistent with the frequentist method. The Bayesian approach also produced smaller standard errors and narrower credible intervals compared to the frequentist (MLE) method. Comparison of the deviance values demonstrated that better model fit was achieved through the Bayesian method.

This study has several limitations. First, prediction of mortality risk in this study was based on data that were collected on the first day of ICU admission. One of the problems that were encountered in the data collection process was missing data. Patients with missing or incomplete data were excluded from the study, giving rise to an overall smaller data set. In addition, data collection was not performed at equal-time intervals for all physiological variables. Routine variables that were easily available were collected more frequently than other variables that required laboratory assessments. Differences in the data collection intervals probably influenced the choice of worst values for the physiological variables and affected prediction accuracy of the models to some extent. Another concern of this study was that the proposed models were all developed based on a single-centre setting. This restricts generalization of the model to other ICUs, unless they share similar patient characteristics and clinical settings as HSA ICU.

## Conclusion

In this study, we applied Bayesian MCMC approach in establishing four predictive models for patients who were admitted to a Malaysian ICU. All four models had good discrimination and calibration in predicting mortality risk in the Malaysian ICU. Model M1 was chosen as the model with the best overall performance and will be used as the future reference model in HSA ICU. This model contained seven variables (age, gender, APS, absence of GCS score, mechanical ventilation, presence of chronic health and ICU admission diagnoses) that are readily available in any intensive care unit setting. This study has also successfully demonstrated application of a Bayesian MCMC approach as an alternative to the traditional frequentist approach.

## Supporting Information

S1 File Relevant data set for this study. (XLSX)
